# A Pyridine Diketopyrrolopyrrole-Grafted Graphene Oxide Nanocomposite for the Sensitive Detection of Chloramphenicol by a Direct Electrochemical Method

**DOI:** 10.3390/nano13030392

**Published:** 2023-01-18

**Authors:** Lingpu Jia, Juan Hao, Long Yang, Jun Wang, Lijuan Huang, Kunping Liu

**Affiliations:** 1 Key Laboratory of Medicinal and Edible Plants Resources Development of Sichuan Education Department, Institute for Advanced Study, Chengdu University, Chengdu 610106, China; 2 Key Laboratory of Medicinal and Edible Plants Resources Development of Sichuan Education Department, Sichuan Industrial Institute of Antibiotics, School of Pharmacy, Chengdu University, Chengdu 610106, China; 3 State Key Laboratory of Environment-Friendly Energy Materials, School of Materials and Chemistry, Southwest University of Science and Technology, Mianyang 621010, China; 4 School of Biological Food and Environment, Hefei University, Hefei 230601, China

**Keywords:** pyridine diketopyrrolopyrrole, N-GO, chloramphenicol, direct electrochemical sensor

## Abstract

A novel direct electrochemical sensor, based on a pyridine diketopyrrolopyrrole/graphene oxide nanocomposite-modified glass carbon electrode (PDPP/GO/GCE), was developed herein for chloramphenicol (CAP) detection. In this research, PDPP was grafted onto GO by C-N bonds and π-π conjugation, which were synergistically confirmed by Fourier transform infrared spectroscopy and X-ray photoelectron spectroscopy. The morphology study shows that PDPP was uniformly dispersed on the GO in the form of particles. The constructed PDPP/GO/GCE showed the strongest response signal to CAP in the evaluation of electrocatalytic activity by cyclic voltammetry compared to that of GO-modified and unmodified GCE, revealing that the introduction of PDPP can effectively improve the electrocatalytic activity of sensors. Moreover, PDPP/GO/GCE had a noticeable current signal when the concentration of CAP was as low as 0.001 uM and had a wide line range (0.01–780 uM) with a low limit of detection (1.64 nM). The sensor properties of the as-obtained PDPP/GO/GCE involved anti-interference, reproducibility, and stability, which were also evaluated and revealed satisfactory results.

## 1. Introduction

Chloramphenicol (CAP), a broad-spectrum antibiotic with strong bactericidal ability, can inhibit protein synthesis in bacterial cells. Therefore, it is often used in the treatment of infectious diseases caused by bacteria [1,2]. However, with wide application in aquaculture and clinical medicine due to its low cost and availability, a large number of CAP residues continually enter water through pharmaceutical wastewater, aquaculture wastewater, hospital wastewater, and domestic sewage, which affects the growth of microorganisms in the environment and even causes dysregulation of the body’s normal flora [3,4,5,6]. At present, many countries have declared their opposition to the use of CAP, while the European Union has allowed usage to be kept below 0.3 µg/kg in food [7]. As a consequence, researchers are constantly trying to seek a rapid, simple, and reliable analytical method for CAP detection at low concentrations in environmental and biological samples [8,9].

According to incomplete statistics, a large amount of published research works on the analysis of CAP have investigated various techniques, such as gas chromatography-mass spectrometry [10], high-performance liquid chromatography [11], Raman scattering [12], capillary electrophoresis [13], chemiluminescence [14], enzyme-linked immunosorbent assay [15], and electrochemical methods [16]. Of these many methods, the electrochemical method is most suitable for CAP routine monitoring due to its miniaturization practicality, low cost, high sensitivity, and selective response. However, it is always a great challenge to construct a fast and highly sensitive electrochemical sensor, which depends on the performance of the modified electrode [17].

In the construction of various electrochemical sensors, graphite oxide (GO) seems to have become an integral part of the composition because of the large number of carboxyl and hydroxyl functional groups and its unique physical and chemical properties [18,19]. However, electrochemical sensors constructed by GO alone have no significant advantages in terms of sensitivity, detection limit or detection range [20]. Hence, GO is often modified by various nanomaterials to enrich the catalytic activity and sensor comprehensive properties [21], such as noble metals, binary metal compounds, metallic oxides, organics, and quantum dots. For CAP sensors based on GO, the modification materials include ZnO [22], Eu_2_O_3_ [23], Co_3_O_4_ [24], and so on, but organics as doped materials are rare.

Pyridine diketopyrrolopyrrole (PDPP), a molecule with a central symmetry, has a conjugated planar backbone and secondary amino, which can prompt the formations of strong π−π interactions and even intermolecular hydrogen bonding with other chemicals [25]. Simultaneously, the outstanding high charge carrier mobility, environmentally friendly nature, and ultra-high corrosion resistance of PDPP have led to its wide use in solar cells, organic field-effects [26], and lithium batteries [27]. However, more attention should be paid to the solubility of PDPP, which can only be slightly dissolved in strong acid or dimethyl formamide at room temperature [28]. Based on the above performance, if PDPP could be extended to a combination of two-dimension nano-materials, it might create new opportunities in the field of electrochemistry [29].

For the limitation of PDPP solubility, there are few materials that can stably exist in strong acid solution or disperse well in DMF. However, it is worth noting that GO as a carbon nanomaterial with special physical and chemical properties can overcome the above problems. Moreover, the carboxyl and hydroxyl functional groups of GO lay the foundation for the combination by chemical bonding, hydrogen bonding or intermolecular forces [30], which have been proved by organic field-effect transistor and organic photovoltaic devices rather than sensors [26,31]. As a result, we propose the synthesis of a PDPP/GO composite in sulfuric acid solution. In addition, the introduction of PDPP inevitably changes its surface properties or electron distribution, which could create a new opportunity for CAP detection.

Considering the importance of CAP determination and the unique superior performances of PDPP and GO, this paper reports a novel composite of PDPP/GO to detect CAP by the direct electrochemical method, which was synthesized by a simple one-step hydrothermal process. The infrared spectrum and X-ray energy spectrum analysis confirmed that PDPP binds strongly with GO by chemical bonds, resulting in organic-N grafted GO composites. At the same time, the PDPP/GO composite-modified glassy carbon electrode (PDPP/GO/GCE) shows more significant electrocatalytic activity for CAP than pure GO, and the involved sensitivity, anti-interference, reproducibility, and stability of the PDPP/GO/GCE sensor have also been evaluated and discussed.

## 2. Materials and Methods

### 2.1. Materials and Instruments

Graphite powder was obtained from Xiya Chemical Technology (Linyi, China). Pyridine diketopyrrolopyrrole (PDPP) was obtained from the laboratory of Chengdu Zhongjin Pharmaceutical Technology Co., Ltd. (Chengdu, China), and the synthesis process is shown in detail in the supporting information. Chloramphenicol (CAP) was obtained from Sichuan Puxiao Standard Material Technology Co., Ltd. (Chengdu, China). Al_2_O_3_ powder, potassium ferricyanide, standard calomel electrode Hg/HgCl_2_ (reference electrode), and platinum wire (auxiliary electrode) were all obtained from Shanghai Chenhua Instrument Co., Ltd (Shanghai, China). The other materials involved in the experiment were analytically pure. Milk was obtained from a local supermarket.

Field-emission scanning electron microscopy (FE-SEM) (Carl Zeiss SMT Pte Ltd., Oberkochen, Germany), X-ray diffraction (XRD) (D/max-r A type Cu Kα, 3–80°), Fourier transformation infrared spectroscopy (FT-IR) (Spectrum one, MA, USA), and X-ray photoelectron spectroscopy (XPS) were used to explore the morphology and structure composition of PDPP/GO. All electrochemical tests were performed with a CHI 832C workstation (CH Instrument, USA) with a three-electrode system, in which the prepared PDPP/GO modified glassy carbon electrode (GCE) played the role of a working electrode.

### 2.2. Preparation of the PDPP/GO Composite

Graphene oxide (GO) was prepared by the Hummers method [32], and the as-prepared GO (0.1002 g) was sonicated for 2 h in ultrapure water (45 mL) to obtain a homogeneous suspension. Second, PDPP (0.0200 g) was dissolved in concentrated sulfuric acid (5 mL) and then slowly added to the GO dispersion. Then, the mixed solution was placed in a stainless Teflon liner and heated at 150 °C for 2 h. The precipitate was filtered and washed successively by 6 M H_2_SO_4_ and ultrapure water to remove unreacted PDPP and dried at 60 °C for 24 h. The obtained composite material was marked as 20% PDPP/GO. Other proportions of PDPP/GO composites (10%, 15%, 20%, 25%) were also obtained following the above method.

### 2.3. Construction of the PDPP/GO/GCE-Modified Electrode

Before use, GCE was polished with 0.05 μm Al_2_O_3_ powder and washed in ethanol and deionized water in an ultrasonic cleaning instrument. Then, 10 uL PDPP/GO ethanol dispersion (2.5 mg/mL) was dropped on the surface of GCE and left to dry naturally. For the comparison, the GO/GCE was prepared by the same method. Before electrochemical measurement, the electrolytic solution was fed with nitrogen for 10 min to remove oxygen.

## 3. Results and Discussion

### 3.1. Morphology and Structure Characterization

**Figure 1:**
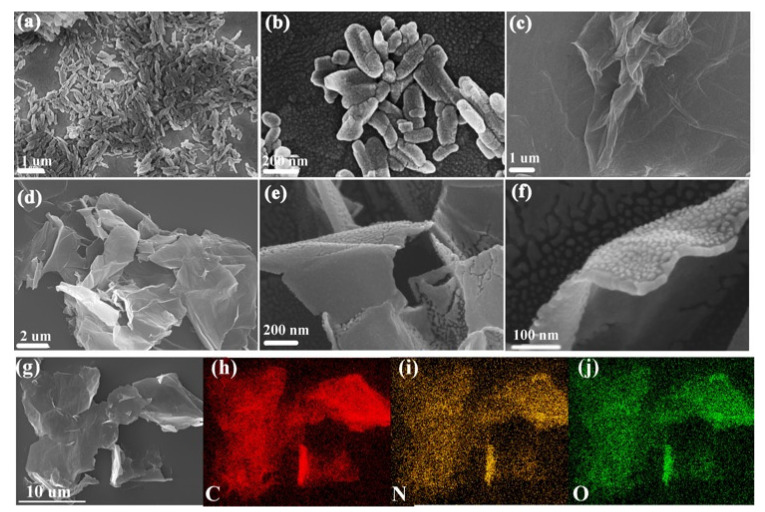
SEM images of PDPP (**a**,**b**), GO (**c**), and PDPP/GO (**d**–**g**). The element mapping images of PDPP/GO with C (**h**), N (**i**), and O (**j**) elements.

The surface morphology of the related materials is presented in Figure 1. The unique grainy morphology with a length of about 100–300 nm corresponds to PDPP, which is the crystalline state of PDPP (Figure 1a,b). After being dissolved in sulfuric acid, PDPP reacted with GO and uniformly attached to the surface of the GO sheets (Figure 1d), leading to an increase in the thickness of GO, as clearly indicated in Figure 1e. Simultaneously, it is worth noting that PDPP no longer existed as rice grain-like particles but as smaller particles in Figure 1f (smaller than 50 nm), which may be due to the possible chemical reaction between PDPP and GO or the solvent effect of PDPP. The chemical composition of the PDPP/GO was analyzed by an energy-dispersive spectrometer (Figure S1), and the spectrum clearly indicated the presence of C, N, and O elements. Moreover, element mapping of PDPP/GO offered a satisfactory dispersion corresponding to the C, N, and O elements (Figure 1g–j), illustrating that PDPP was evenly distributed on the lamellar structure surface of GO.

**Figure 2:**
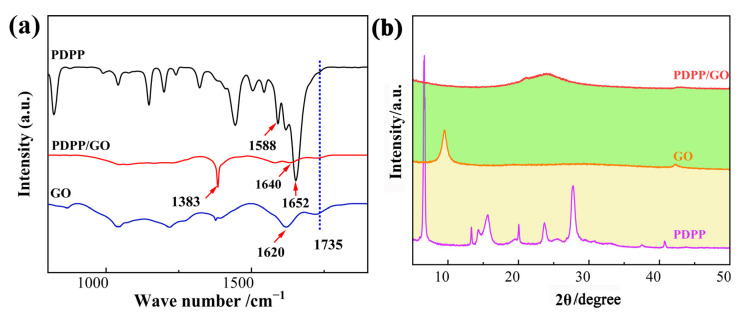
FT-IR (**a**) and XRD (**b**) spectra of GO, PDPP, and PDPP/GO.

The reaction mechanism and structure of the PDPP/GO composite were analyzed by infrared spectroscopy, as displayed in Figure 2a. In the curve of PDPP, the peaks at 1735, 1652, and 1588 cm^−1^ are ascribed to the stretching vibrations of C=O, C=C, and C=N, respectively. The characteristic absorption peaks corresponded to C=O and C=C of GO located at 1735 and 1620 cm^−1^ [33]. After the reaction, the peak of C=O (1735 cm^−1^) remained stable and the C=C peak occurred at 1640 cm^−1^, which may be due to π-π conjugation or the synergistic contribution of functional groups. The emergence of a strong peak at 1383 cm^−1^ in the curve of PDPP/GO was classified as the stretching vibration of C-N, revealing that PDPP integrates with GO by strong C-N chemical bonding [34]. Figure 2b shows the structural change of the related materials before and after the reaction. Initially, PDPP had a good crystal structure with some strong diffraction peaks at 6.6°, 13.3°, 15.6°, 19.9°, 23.6°, and 27.7°. For GO, a spiked peak at 9.5° was identified as the characteristic diffraction peak of GO, and the peak at 43° was ascribed to (100) basal-spacing. However, a new diffraction peak appeared at 24.2° for the PDPP/GO composite, while there was no change in the (100) peak, indicating that GO was reduced to graphene oxide with different kinds of defects [35]. Furthermore, no crystal peak of PDPP was observed, which suggests that the crystalline structure of PDPP was destroyed.

**Figure 3:**
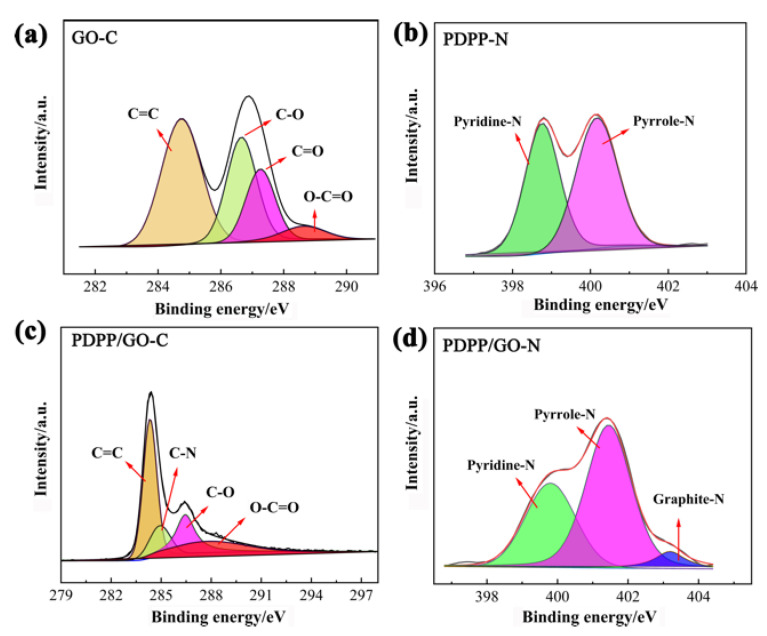
XPS spectrum of GO with C1s (**a**), PDPP with N1s (**b**), and PDPP/GO with C1s (**c**), N1s (**d**).

**Figure 4:**
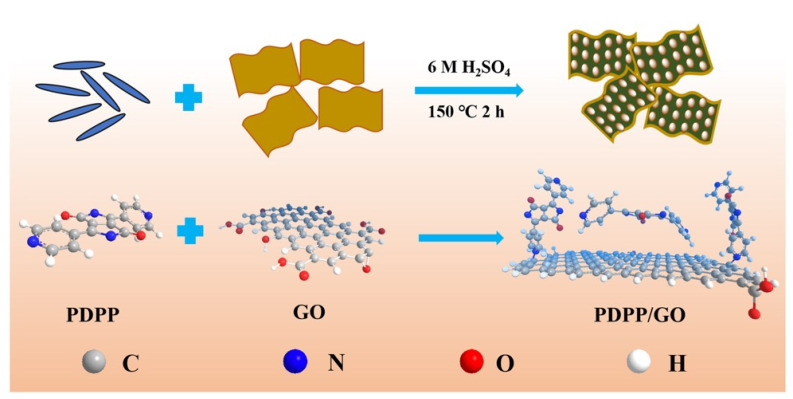
The reaction and structure diagrams of PDPP/GO.

XPS analysis was used to further investigate the distribution of different elements and the structure of PDPP/GO. Figure 3a shows the high-resolution C1s spectra of GO, and four peaks at 284.8 eV, 286.6 eV, 287.3 eV, and 288.6 eV were defined as C=C, C-O, C=O, and O–C=O, respectively. The high-resolution of N1s in Figure 3b shows two peaks at 398.8 and 400.1 eV, which belong to the pyridine-N and pyrrole-N of PDPP [36], respectively. When PDPP reacted with GO, the C1s spectra of PDPP/GO showed that the binding energies decreased to 284.3 eV (C=C), 286.5 eV (C=O), and 288.1 eV (O-C=O) compared to GO. A new C-N peak appeared at 285.0 eV (Figure 3c) [37]. Similarly, Figure 3d displays an extra graphite-N at 403.1 eV, except that the pyridine-N and pyrrole-N shifted to higher binding energies of 399.8 and 401.4 eV, respectively, compared with PDPP [38]. These results corroborate each other and suggest the formation of a new graphite-N bond between GO and PDPP, which is also in agreement with the FT-IR spectra analysis. Furthermore, the negative shift to GO and positive shift to PDPP together indicate that the entire electron cloud of PDPP moved toward GO, which was due to the formation of graphite-N and π-π conjugation. In the PDPP molecule, the theoretical ratio of pyridine-N to pyrrole-N is 1:1, which was verified by the peak area in Figure 3b. For PDPP/GO, the peak area of pyridine-N decreased, and it is worth noting that the sum of the peak areas of pyridine-N and graphite-N was close to that of pyrrole-N. Therefore, we conclude that (1) the molecular structure of PDPP was not broken; (2) PDPP bonded to GO through pyridine-N; and (3) there was a π-π conjugated interaction between PDPP and GO. Hence, the structure and reaction of PDPP/GO can be determined, as shown in Figure 4.

### 3.2. Electrochemical Test for Different Electrodes Regarding CAP

**Figure 5:**
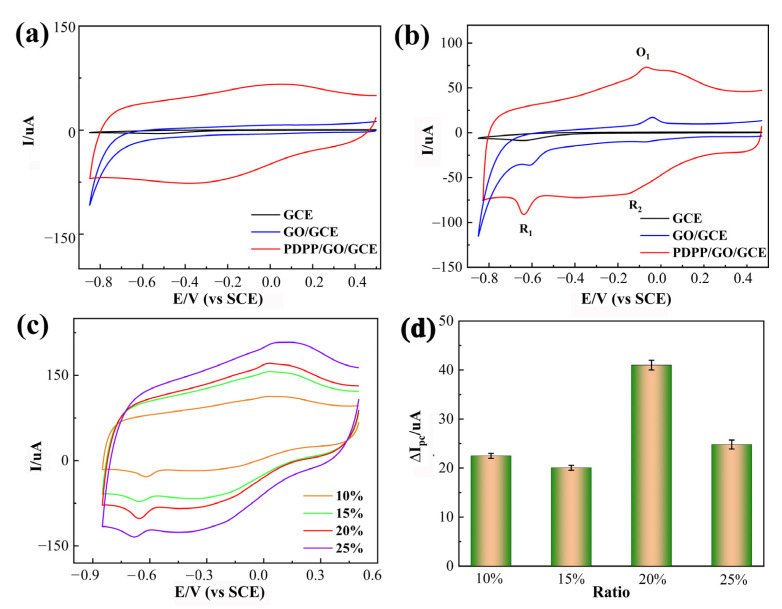
CVs of different electrodes (GCE, GO/GCE, PDPP/GO/GCE) in 0.1 M PBS (pH 6.5) without (**a**) and with (**b**) 0.01 mM chloramphenicol; (**c**) CVs of PDPP/GO/GCE at different ratios, 10%, 15%, 20%, and 25%, in 0.1 M PBS (pH 6.5) containing 0.01 mM chloramphenicol; (**d**) absolute peak reduction current (∆I) vs. the ratio from the CVs. All scan rates: 50 mV/s.

To evaluate the electrochemical performances of bare GCE, GO/GCE, and PDPP/GO/GCE, the electrocatalytic behavior was determined in 0.1 M phosphate buffer solution (PBS, pH 6.5) by cyclic voltammetry (CV) at a scan rate of 50 mV/s. In the range of −0.85–0.5 V, there was no obvious redox peak in all CV curves without chloramphenicol (Figure 5a), indicating that these electrodes only conduct electrons rather than undergo electrochemical reactions under the above conditions. After the addition of 0.01 mM CAP (Figure 5b), the bare GCE exhibited an inconspicuous reduction peak at −0.643 V with a small cathodic peak current (*I_pc_*, 8.6 μA), which manifested as a slow electrochemical reduction of CAP at the bare GCE. Similarly, when GCE was modified by GO, the reduction peak was observed at −0.613 V (35.6 uA) with a marked *I_pc_* increase and a positive shift of the cathodic peak potential (*E_pc_*) compared with the bare GCE. The results show that GO had better electrochemical activity toward CAP. Meanwhile, a pair of redox peaks appeared at −0.041 V and −0.085 V. Interestingly, the PDPP/GO/GCE also exhibited a significant reduction peak (−0.633 V, 92.5 uA) and a pair of redox peaks (−0.073 V, 72.8 uA; −0.136 V, 67.1 uA). Based on the above CV curves, PDPP/GO/GCE showed the highest cathodic peak currents and the lower reduction peak potential, proving that PDPP-doped GO can enhance electrocatalytic activity towards the reduction of CAP.

**Figure 6:**
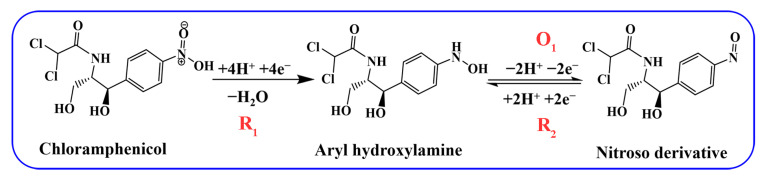
Electrochemical mechanism of CAP at PDPP/GO/GCE.

In the CV curve of electrocatalytic CAP at PDPP/GO/GCE, the reaction of R_1_ can be ascribed to four electrons and four protons in a reduction process in which the nitro group of CAP is reduced to aryl hydroxylamine [22,39]. These redox peaks at O_1_ and R_2_ correspond to the two electron and two proton transfer mechanism between the nitroso group derivative and hydroxylamine [40], as displayed in Figure 6. Different doped ratios of PDPP were also investigated by CV in Figure 5c. As the PDPP increased, 20% PDPP/GO/GCE showed the best catalytic activity for CAP with the largest absolute peak reduction current (∆*I_pc_* = 18.9 uA) in Figure 5d, so the 20% PDPP/GO/GCE was used for subsequent tests and labeled PDPP/GO/GCE.

The electrochemically active surface area (ECSA) is a very important factor affecting the electrode surface reaction, and it was analyzed by the cyclic voltammetric technique in the non-Faradaic region from 0.07 V to 0.27 V in 0.1 M PBS containing 0.01 mM CAP at various scan rates (20–120 mV/s), as shown in Figure S2a. The slope (2.32 mF/cm^2^) of the linear regression plot corresponded to C_dl_ (Figure S2b). The ECSA was calculated as 58 cm^2^ from the following equation: ECSA = C_dl_/C_s_ [41], where C_s_ is the C_dl_ of an ideal flat electrode (for GCE, Cs = 0.04 mF/cm^2^).

### 3.3. Effect of the pH and Scan Rate

**Figure 7:**
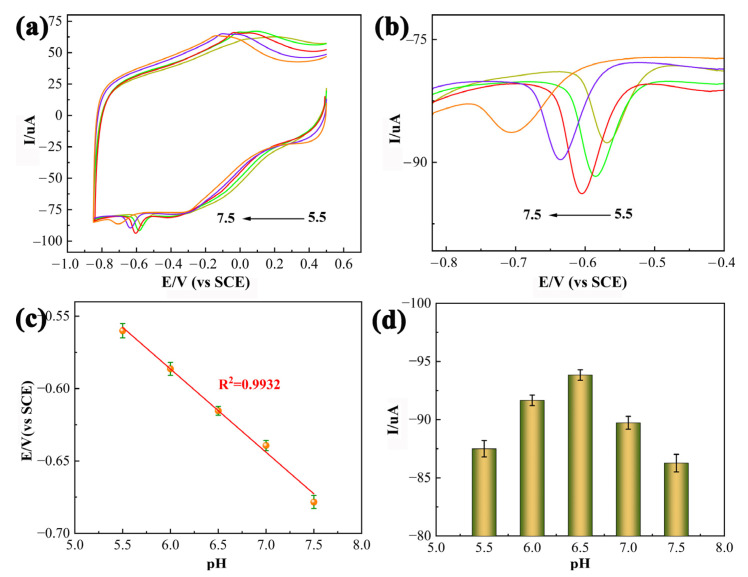
(**a**) CV curves of 0.01 mM CAP at the PDPP/GO/GCE at various pH values from 5.5 to 7.5 and an enlarged view (**b**), scan rate: 50 mV/s; (**c**) linear relationship of E_Pc_ versus the pH value; (**d**) the plot of *I_pc_* versus the pH value.

The pH value of the electrolyte had a significant influence on the electrochemical behavior of CAP, so the electrochemical reaction of 0.01 mM CAP at the PDPP/GO/GCE was investigated by CV in 0.1 M PBS with different pH values (from 5.5 to 7.5). As shown in Figure 7a, the *E_pc_* (R_1_) of CAP shifted negatively when the pH value increased (Figure 7b), revealing that protons participated in the reduction reaction of CAP. The linear regression equation between *E_pc_* and pH is established by Equation (1):

(1) $$E_{pc}=-0.0578pH-0.2401(R^{2}=0.9932)$$

The equation expresses a slope value of 0.0578 V/pH (Figure 7c), which matches the theoretical value of 0.0591 V/pH and demonstrates that the reduction process of the nitro group of CAP to hydroxylamine at the PDPP/GO/GCE is accompanied by an equal number of protons and proton transfer [42]. Figure 7d shows the plot of *I_pc_* versus the pH value. The *I_pc_* of CAP increased until the pH value increased to 6.5 and then decreased. Hence, the optimal pH value of PBS was 6.5 for the electrocatalytic reduction of CAP at the PDPP/GO/GCE.

**Figure 8:**
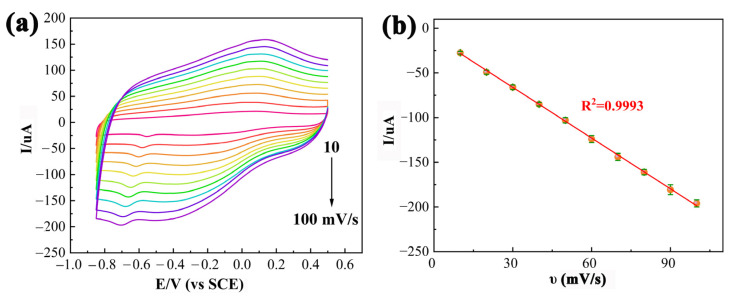
(**a**) CVs of PDPP/GO/GCE in 0.1 M PBS containing 0.01 mM CAP at various scan rates (10–100 mV/s), (**b**) the plots of peak current vs. υ.

The effect of the scan rate (υ) on the PDPP/GO/GCE was also studied by CV with various scan rates from 10 to 100 mV/s in 0.1 M PBS (pH 6.5) containing 0.01 mM CAP (Figure 8a). As the scanning rate increased, both the *I_pc_* and *E_pc_* of CAP (R_1_) gradually increased. In addition, the *I_pc_* was proportional to the scan rate based on the linear equation, Equation (2), as displayed in Figure 8b.

(2) $$I_{pc}=-1.887v-9.9667(R^{2}=0.9993)$$

Hence, in can be deduced that the electrochemical action of CAP at the PDPP/GO/GCE was a typical adsorption-controlled process [43,44].

### 3.4. Determination of CAP

**Figure 9:**
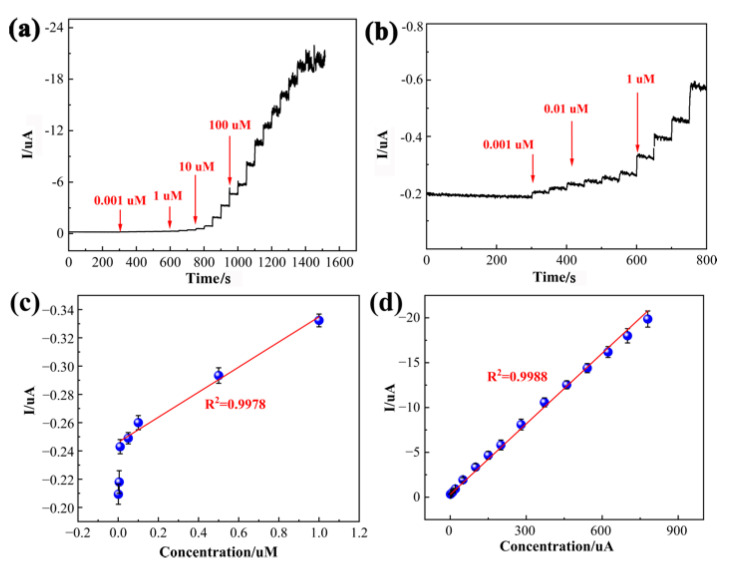
Current–time curve of the PDPP/GO/GCE at −0.62 V for successive additions of different concentrations of CAP in stirred PBS (pH 6.5) (**a**) and an enlarged view (**b**); (**c**) The calibration curve of the low concentration range (0.01–1 uM) and (**d**) high concentration range (1–780 uM) obtained from the amperometric response.

Under optimal conditions, the amperometric (i-t) response at a fixed potential of −0.62 V was determined to evaluate the sensing performance of PDPP/GO/GCE with a time interval of 50 s. As displayed in Figure 9a, the i-t curve showed a stable and well-defined amperometric response with the addition of CAP, even at a low concentration in the enlarged view in Figure 9b. A steady current was achieved within 3 s, reflecting the rapid response of PDPP/GO/GCE. Figure 9c,d shows the relevant calibration curve between the concentration of CAP and the current, and the PDPP/GO/GCE responded linearly to CAP from 0.01 to 780 uM with equations of I = −0.0895C − 0.2445(R^2^ = 0.9978) (0.01–1 uM) and I = −0.0251C − 0.6682(R^2^ = 0.9988) (1–780 uM). The limit of detection (LOD), 1.64 nM, can be calculated by 3 σ/S, where σ represents the standard deviation of the intercept of the regression line and S is the slope of the calibration curve. Compared to the published CAP sensors in Table 1, it is noteworthy that the as-prepared PDPP/GO/GCE sensor had superior sensing performance in either the linear range or at the LOD.

### 3.5. Selectivity, Reproducibility, and Stability

**Figure 10:**
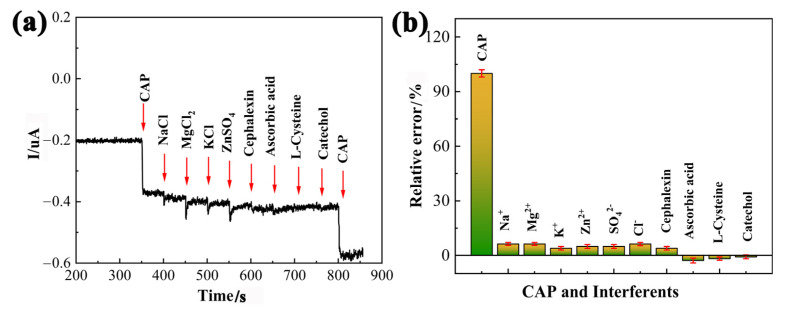
Interference investigation of the PDPP/GO/GCE by i-t test technique (**a**) and histogram of the selectivity toward interferents (**b**).

The selectivity of the PDPP/GO/GCE to detect CAP was evaluated by measuring the current at a fixed potential in the presence of potential interferents (Figure 10a). In the test, 100-fold concentrations of inorganic ions, including Na^+^, Mg^2+^, K^+^, Zn^2+^, SO_4_^2−^, and Cl^−^, were added to 0.1 M PBS (pH 6.5) with 5 uM of CAP, and the results reveal that the PDPP/GO/GCE quickly and significantly responded to CAP rather than the above interferents. Furthermore, cephalexin, ascorbic acid, L-cysteine, and catechol with concentration of 50 uM were evaluated, which had no significant effects on the CAP signal. Figure 10b shows the relative error caused by different interferents in the detection of CAP, and the current signal of CAP at the PDPP/GO/GCE remained stable even in the presence of high concentrations of interferents. These results indicate that the constructed sensor has outstanding anti-interference performance.

The reproducibility of the PDPP/GO/GCE was investigated by six similarly modified electrodes at 0.62 V under the optimal conditions towards 5 uM CAP. The obtained relative standard deviation (RSD) with 4.96% of the current response indicates that the proposed sensor has good reproducibility. As for the stability pact, about 93.8% of the initial response current was achieved by intermittently measuring the current response over a storage period of 15 days, suggesting the excellent long-term stability of PDPP/GO/GCE. Hence, the PDPP/GO/GCE sensor has satisfactory comprehensive performance for CAP detection.

### 3.6. Real Sample Analysis

In order to verify the practical application of the sensor, real sample analysis was investigated in tap water and milk, which were filtered by a Millipore filter membrane (0.45 μm). The collected samples were diluted with 0.1 M PBS (pH 6.5) from 500 uL to 6.0 mL and then tested by the standard addition method with CAP at −0.62 V. Table 2 lists the samples designed at low, moderate, and high concentrations, and all recoveries remained between 93.8% and 108.3% with RSDs of 1.9–4.6%, indicating that the determination of CAP at the PDPP/GO/GCE was highly effective in the real samples. The PDPP/GO/GCE sensor was also validated by high-performance liquid chromatography and showed satisfactory results, as indicated in Figure S3 and Table S1.

## 4. Conclusions

In this study, a sensitive CAP direct electrochemical sensor was constructed by functionalized GO with conductive conjugated PDPP. Through structure, morphological, and compositional characterizations of the PDPP/GO composite, the following results were obtained: (1) the molecular structure of PDPP was not broken; (2) there was a strong π-π conjugate interaction between PDPP and GO; (3) GO connected to the pyridine-N of PDPP to form a new C-N chemical bond; (4) GO was reduced after the reaction; and (5) the morphology of PDPP changed from rice granular to a small particle (50 nm) with uniform distribution on the surface of GO. The synthesized PDPP/GO composite-modified GCE had more active sites and better electrical conductivity, which were proven in the electrocatalytic activity test for CAP by CV. Moreover, the ratio of the PDPP/GO composite and the test condition of CAP were also optimized. The subsequent sensor performance displayed a wide linear range (0.01–780 uM), a low LOD (1.64 nM), as-expected anti-interference, reproducibility, and stability. Satisfactory results were also obtained in the test of actual samples, which one would expect to be produced and used for sensitive detection of CAP in the future.

## Figures and Tables

**Table 1:** Evaluation of CAP based on various modified electrodes.

Modified Electrode	Method	Linear range (uM)	LOD (uM)	Ref.
3DRGO/GCE	DPV	1–113	0.1500	[45]
NiCo_2_O_4_@C/GCE	DPV	0.5–320	0.0352	[46]
MoN@S-GCN/GCE	DPV	0.5–2450	0.0069	[47]
GO/SmMoSe_2_/GCE	LSV	0.01–244	0.0050	[48]
g-C_3_N_4_/MnWO_4_/GCE	DPV	0.004–0.071	0.0013	[49]
Co_3_O_4_@rGO/GCE	i-t	0.1–1500.0	0.1000	[24]
PDPP/GO/GCE	i-t	0.01–780	0.0016	This work

**Table 2:** Determination of CAP in tap water and milk samples.

Samples	Detected (μM)	Added (μM)	Found (μM)	Recovery (%)	RSD (%)
Tap water	0	0.1	0.0938 ± 0.002	93.8	1.9
	0	10	10.55 ± 0.33	105.5	3.1
	0	100	99.3 ± 1.79	99.3	1.8
Milk	0	0.1	0.0965 ± 0.004	96.5	4.1
	0	10	10.83 ± 0.49	108.3	4.6
	0	100	105.5 ± 4.75	105.5	4.5

## Data Availability

Not applicable to this article.

## Supplementary Materials

The following supporting information can be downloaded at: https://www.mdpi.com/article/10.3390/nano13030392/s1, Synthesis of PDPP, Figure S1: EDS spectrum of PDPP/GO, Figure S2: (a) The CV responses were measured in the non-Faradaic region from 0.07 V to 0.27 V for PDPP/GO/GCE in 0.1 M PBS containing 0.01 mM CAP at various scan rates (20–120 mV/s). (b) Linear plot of Δj/2 at a 0.17 V vs. scan rate, Figure S3: The calibration curve of the low concentration range of CAP (0.1, 0.5, 1, 5, 10, 50, 100 ug/L), Table S1: Determination of CAP in tap water sample by high-performance liquid chromatography.
