# Supplementary material for: A Pyridine Diketopyrrolopyrrole-Grafted Graphene Oxide Nanocomposite for the Sensitive Detection of Chloramphenicol by a Direct Electrochemical Method
Source: Nanomaterials (Basel). 2023 Jan 18;13(3):392. doi: 10.3390/nano13030392 (PMC9921031; doi:10.3390/nano13030392)
Supplement: Supplementary file 1 [file nanomaterials-13-00392-s001.zip › nanomaterials-2154815-supplementary.pdf]

Supplementary Materials

A Pyridine Diketopyrrolopyrrole-Grafted Graphene Oxide Nanocomposite for the Sensitive Detection of Chloramphenicol by a Direct Electrochemical Method

Synthesis of PDPP

Sodium metal (0.8 g) was added to tert-amyl alcohol (30.0 ml) and dissolved completely in 115℃ to form the sodium tert-amylate solution. To this solution, 3-cyanopyridine (0.2 g) was added slowly, followed by the solution of diethyl succinate (0.2 g) in tert-amyl alcohol (10.0 ml) drop by drop. After reaction under 115 °C for 3 h more, methyl alcohol (80 ml) and concentrated HCl (5 ml) were added to give precipitate. The filter cake was washed by methyl alcohol (100 ml), H_2O (100 ml) and methyl alcohol (100 ml) in sequence. Then the filter cake was suspended in dimethyl sulfoxide (20 ml) under heating of 100 °C for 48 hours. Again, the filter cake was washed by methyl alcohol (100 ml), H_2O (100 ml) and methyl alcohol (100 ml) in sequence, and dried under 40 °C in vacuum for 4 hours to obtain the brown product with a yield of 80.1%.

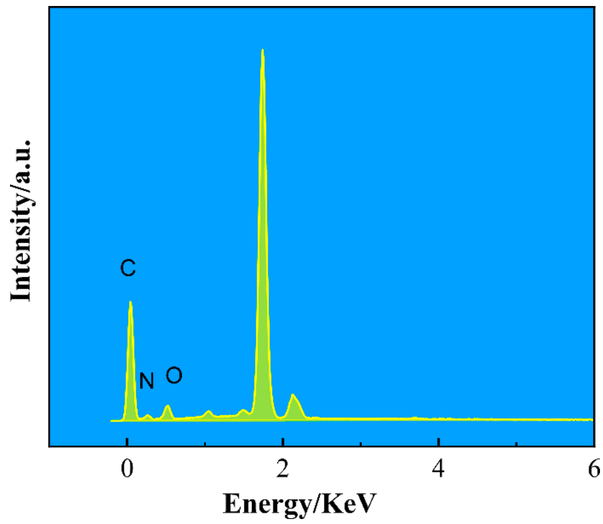

Figure. S1 EDS spectrum of PDPP/GO.

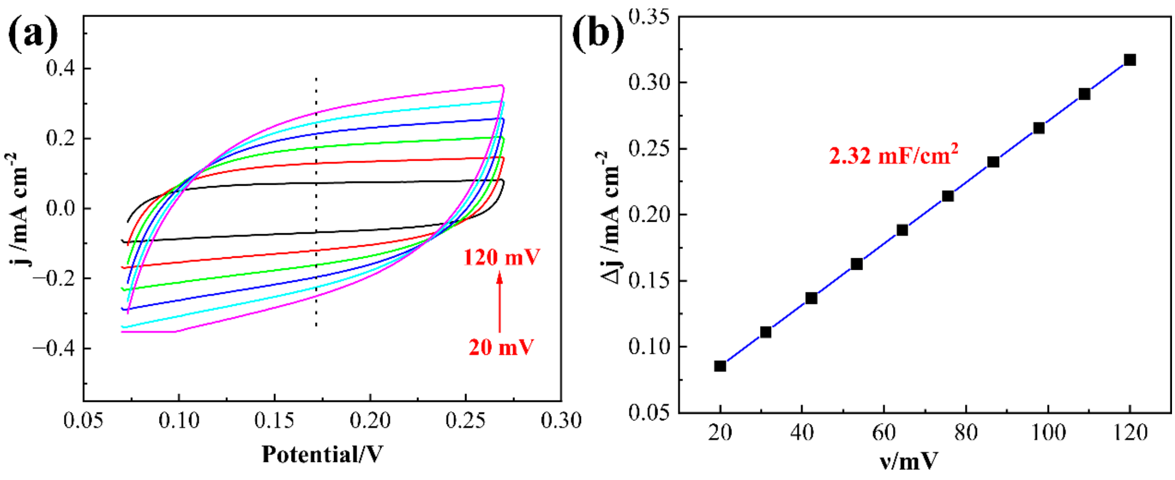

Figure. S2 (a) The CV responses were measured at non-Faradaic region from 0.07 V to 0.27 V for PDPP/GO/GCE in in 0.1 M PBS contained 0.01 mM CAP at various scan rates (20–120 mV/s). (b) Linear plot of Δj/2 at 0.17 V vs. scan rate.

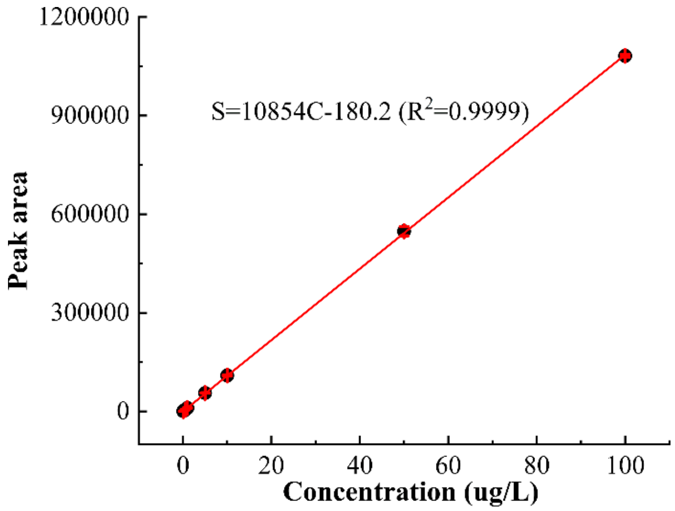

Figure. S3 The calibration curve of low concentration range, CAP (0.1, 0.5, 1, 5, 10, 50, 100 ug/L).

Table S1. Determination of CAP in tap water sample by high performance liquid chromatography.

sample	Added (μM)	Found (μM)	Recovery (%)	Average value	RSD (%)
Tap water	0.1	0.1065	106.5	107.5	1.28
	0.1	0.1070	107.0		
	0.1	0.1091	109.1		
